# Medical Ethics

**Published:** 1858-11

**Authors:** 


### EDITORIAL AND MISCELLANEOUS.

### MEDICAL ETHICS.

It is sometimes a question in our minds whether there is not as decided "*Quackery*" in the "*Regular*" Profession of Medicine as among the hordes of "Irregulars."

It is true that the manifestation is not always exactly the same, but we fear the aim and object, is similar.

While the ignorant (save of the means of gulling the public) and unprincipled "Quack" commends his superior skill, "*particularly* in Obstetrics and the diseases peculiar to females," through the public prints, and vaunts his unequalled success *generally*, in treating all manner of disease; the more Regular Quack by a system of *maneuvreing*, *boasting* and *electioneering* is, in our judgment, not less culpable, however he may be cloaked with nominal orthodoxy or screened by the forbearance of more honorable and meritorious brethren.

The truth is, we have more respect for the independent and defiant empiric, who asks or expects nothing at the hands of the regular profession, than we can have for the individual, who under the code of professional honour, is receiving the countenance and support which the ethics and etiquette of the profession requires to be extended from one member to another, and yet practically disregards the obligations, which he has assumed upon entering into the ranks of an honourable calling.

We are grieved to say that in our judgment, the art of medicine has suffered more from its pretended friends than from open enemies, and we shall not hesitate to express a greater degree of contempt for the mercenary and selfish *intriguer*, who is found in the regular ranks boasting of his *discoveries* or his *peculiar* skill in the treatment of disease—whether in person or through friends—than we have for the lawless "Cossack," who makes unscrupulous, but declared war, upon our science.

Could we expel from our profession or prevent from entering into it, those who love *money* above honour, science and humanity, we should greatly increase the dignity and elevate the character of our calling, besides adding largely to its efficiency in the relief of suffering humanity. So mote it be.

